# Progressive Stroke Caused by Neurosyphilis With Concentric Enhancement in the Internal Cerebral Artery on High-Resolution Magnetic Resonance Imaging: A Case Report

**DOI:** 10.3389/fneur.2021.675083

**Published:** 2021-08-30

**Authors:** Kejia Zhang, Fengna Chu, Chao Wang, Mingchao Shi, Yi Yang

**Affiliations:** ^1^Stroke Center & Clinical Trial and Research Center for Stroke, Department of Neurology, The First Hospital of Jilin University, Changchun, China; ^2^China National Comprehensive Stroke Center, Changchun, China; ^3^Jilin Provincial Key Laboratory of Cerebrovascular Disease, Changchun, China

**Keywords:** stroke, high resolution magnetic resonance imaging, neurosyphilis, meningovascualr syphilis, neurosyphilic arteritis

## Abstract

**Background:** Neurosyphilis can initially present as a stroke. However, the general management strategy for stroke may not be effective for this condition. Intracranial vessel wall imaging indicating arteritis can help differentiate neurosyphilis from other causes of stroke.

**Case presentation:** A 59-year-old Chinese woman presented with an acute infarct in the left basal ganglia and multiple stenoses in the bilateral middle cerebral arteries, anterior cerebral artery, and basilar artery, which aggravated twice, despite antiplatelet treatment. High-resolution magnetic resonance imaging (HR-MRI) suggested concentric enhancement in the left middle cerebral artery. *Treponema pallidum* test results were positive, suggesting neurosyphilis.

**Conclusions:** HR-MRI provides valuable information regarding arteritis, which is helpful in differentiating neurosyphilis from other causes of stroke. Antiplatelet medication should be used judiciously for neurosyphilis-related stroke.

## Introduction

Syphilis, a sexually transmitted disease, is caused by *Treponema pallidum*. Neurosyphilis occurs when *T. pallidum* invades the central nervous system, which may initially present as a stroke (1, 2). For these patients, the general management strategies for stroke, including the use of antiplatelet and anticoagulant agents, may be less effective. Therefore, the identification of neurosyphilis during the early stages of the disease is essential. Apart from serum or cerebrospinal fluid (CSF) findings of *T. pallidum*, high-resolution magnetic resonance imaging (HR-MRI) indicating arteritis can help differentiate neurosyphilis from strokes caused by other factors (3, 4). Here, we present a unique case of progressive stroke caused by neurosyphilis and radiological characteristics of the intracranial vessel wall imaging.

## Case Presentation

**Figure 1:**
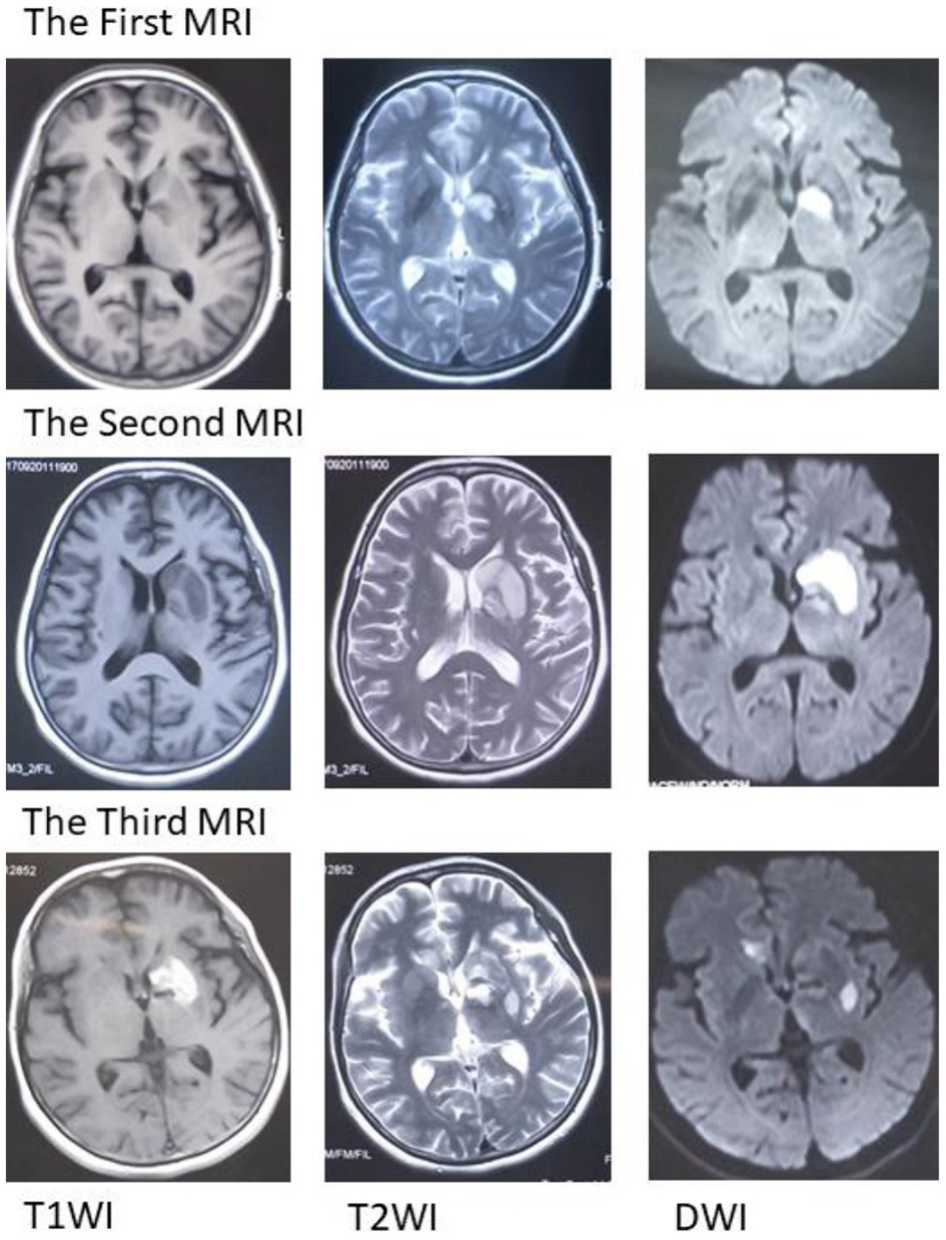
Magnetic resonance imaging (MRI). The first MRI suggests an acute infarct in the left basal ganglia. The second MRI scan suggests an expanded infarct in the left basal ganglia. The third MRI suggests acute infarction in the left basal ganglia and right callosum genu and bleeding in the left basal ganglia. MRI, magnetic resonance imaging; T1WI, T1-weighted imaging; T2WI, T2-weighted imaging; DWI, diffusion-weighted imaging.

A 59-year-old Chinese woman was hospitalized due to bradyglossia and weakness of the right lower limb. She denied smoking, drinking, hypertension, diabetes mellitus, coronary heart disease, and previous stroke. MRI suggested an acute infarct in the left basal ganglia (Figure 1) and the right posterior horn of the lateral ventricle. Aspirin, clopidogrel, atorvastatin, and butylphthalide were initiated based on a diagnosis of ischemic stroke.

**Figure 2:**
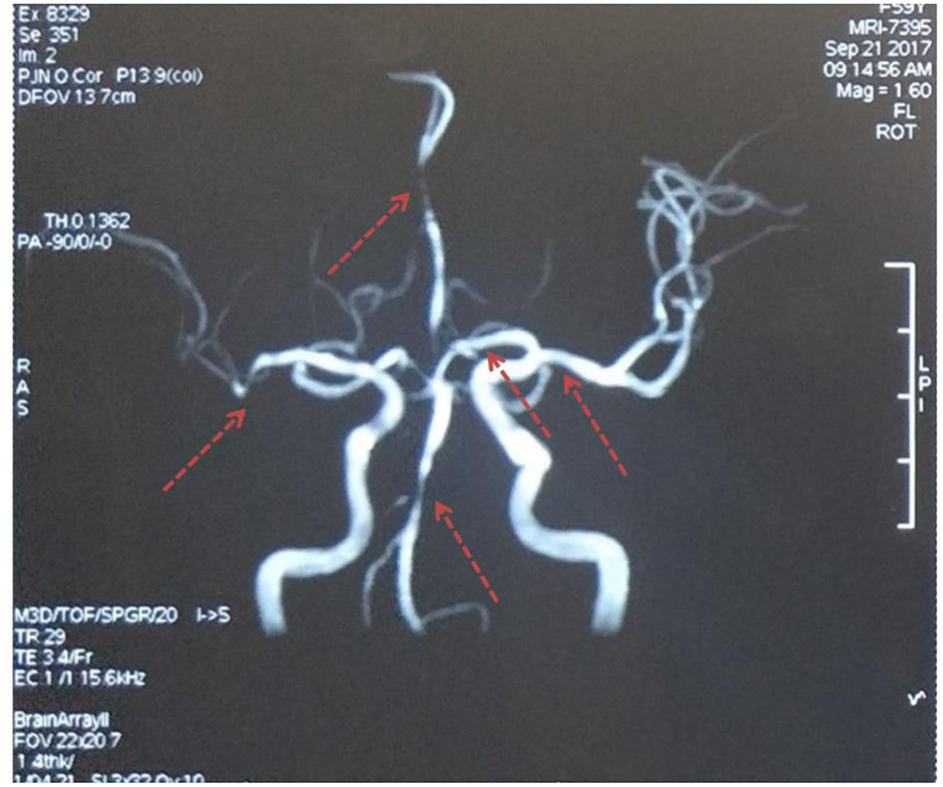
Magnetic resonance angiography. Multiple stenoses in the bilateral middle cerebral arteries, anterior cerebral artery, and basilar artery. The red arrows indicate stenosis.

Unfortunately, her clinical symptoms deteriorated 16 days after disease onset. She could not walk independently and leaned to the right side. Drooping of the right angulus oris was also noted. The patient was then admitted to our stroke center. Neurological examinations identified hemiglossoplegia, prosopoplegia, paraparesis of the right limb (5–/5), bradyglossia, and positive Babinski and Chaddock signs. Muscle tone, deep tendon reflexes, cerebellar signs, sensory abnormalities, and cranial nerves were unremarkable. The National Institutes of Health Stroke Scale score was assessed as 2. A repeat brain MRI suggested expanded infarct lesions in the left basal ganglia (Figure 1). New lacunar infarct lesions in the right corona radiata and stenosis in the bilateral middle cerebral arteries (MCA), anterior cerebral artery (ACA), and basilar artery (BA) were noted (Figure 2). The standard therapy for stroke management, including aspirin, clopidogrel, atorvastatin, butylphthalide, and edaravone, was administered continuously.

Routine serology and hematological tests suggested elevated blood glucose levels with a fasting blood glucose of 6.37 mmol/L, 2 h post-prandial blood glucose of 8.62 mmol/L, and glycosylated hemoglobin level of 6.10%. Blood pressure, serum homocysteine levels, and electrocardiography and echocardiography results were normal. Other risk factors for cerebral vascular disease were not remarkable.

Serum *T. pallidum* particle agglutination (TPPA) was positive, and the rapid plasma reagin assay (RPR) value was 1:16. A lumbar puncture was performed, and the results showed that the CSF was clear with a pressure of 110 mm H_2_O. CSF protein (0.67 g/L, 0.15–0.44) and leukocyte (148 × 10^6^/L, normal 0–8) levels were elevated with a positive Pandy test. CSF TPPA test results were positive, while RPR results were negative. No chancres or any other signs of syphilis were identified. The patient denied promiscuity. Her husband died 5 years ago. However, the patient used to get pedicures.

**Figure 3:**
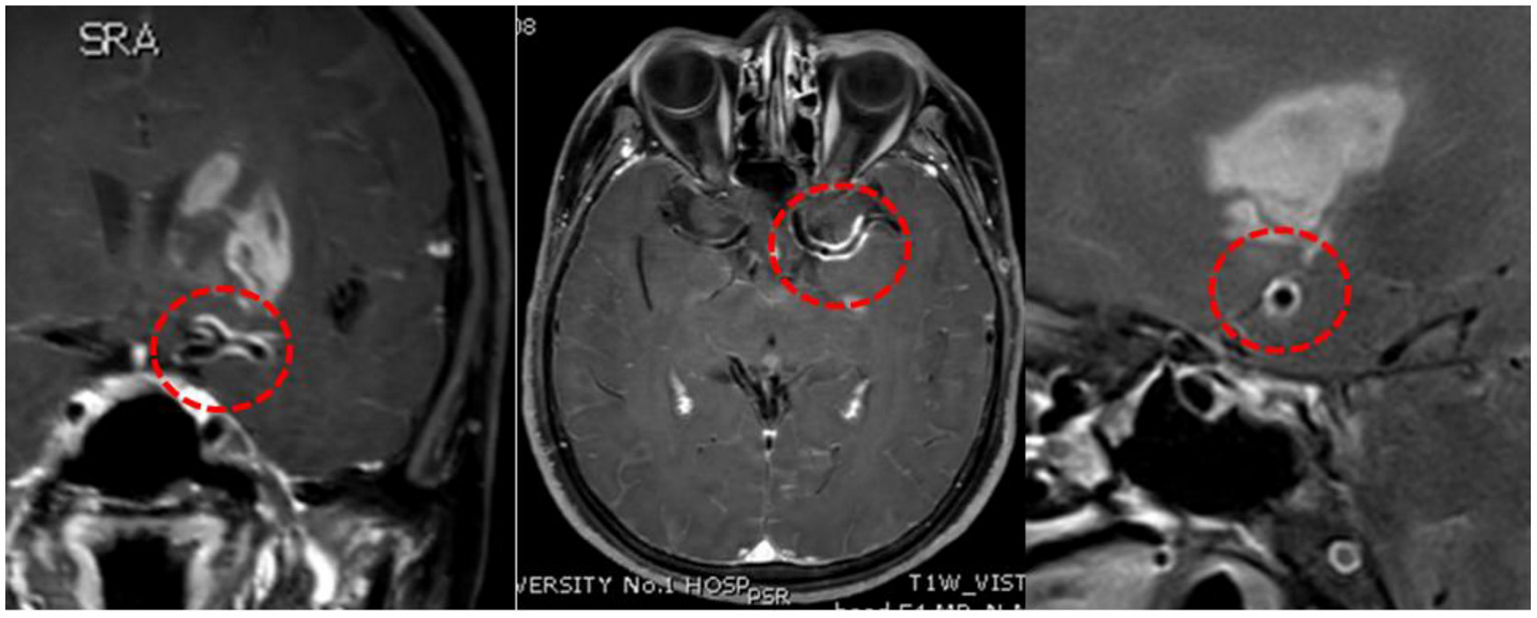
High-resolution magnetic resonance imaging. Concentric enhancement in the left middle cerebral artery and anterior cerebral artery. Red circles suggest vessel wall enhancement.

Intracranial vessel wall imaging with HR-MRI and cognitive scales was performed. Concentric contrast enhancement of the vessel walls was observed in the left MCA and ACA (Figure 3). The enhancement was observed in the entire M1 segment of the left MCA and A1 segment of the ACA, which was uniform, continuous, and similar in intensity. In the contralateral MCA, ACA, and BA, the enhancement was not remarkable. Syphilitic arteritis was thus considered in the left ACA and MCA, and the infarct in the left basal ganglia could be explained accordingly. The Mini-Mental State Examination score was 23/30, and the Montreal Cognitive Assessment score was 17/30. Cognitive impairment, neurological impairment, damage to intracranial arteries, positive CSF TPPA test results, and elevated CSF protein levels and leukocyte counts were identified. Neurosyphilis, as generalized paresis of the insane and meningovascular syphilis, was considered. Antibiotic treatment was initiated. Roxithromycin (500 mg, four times orally per day) was administered as the patient was allergic to penicillin and ceftriaxone. Hexadecadrol was initiated 3 days prior to roxithromycin administration, to prevent the herxheimer reaction.

**Figure 4:**
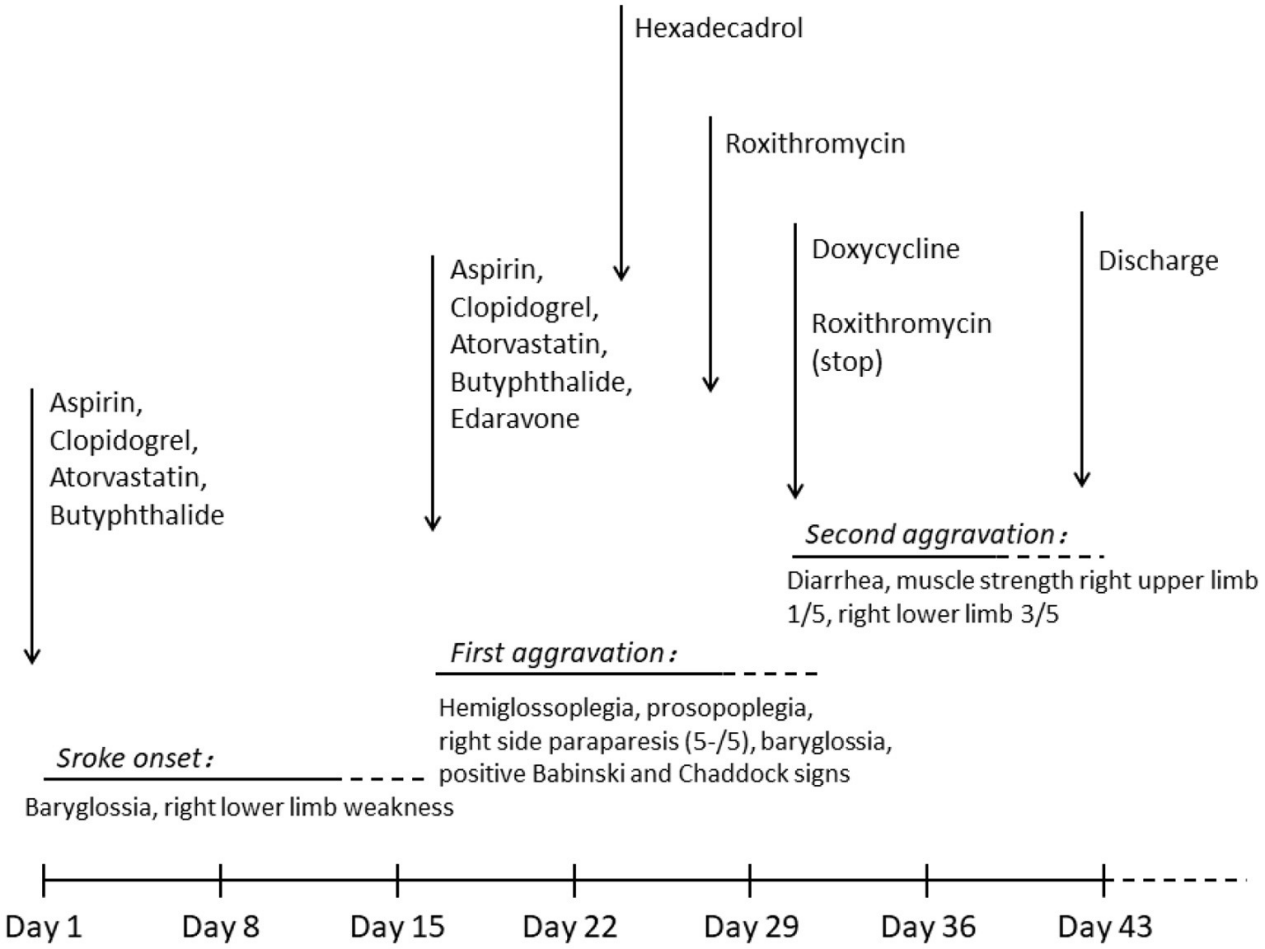
Timeline of stroke aggravation and intervention. Stroke occurred on day 1 and was aggravated twice (days 16 and 30). Hexadecadrol was initiated on day 23, roxithromycin was initiated on day 27, and roxithromycin was replaced with doxycycline on day 30. The patient was discharged on day 41.

On the third day following antibiotic initiation, the neurological function of the patient deteriorated again, which was accompanied by severe diarrhea. Muscle strength of the right side declined with upper limbs measuring one-fifth and lower limbs measuring three-fifths. Her brain MRI suggested acute infarction in the left basal ganglia and right callosum genu and bleeding in the left basal ganglia (Figure 1). Considering that diarrhea may be a side effect of roxithromycin, roxithromycin was replaced by doxycycline (0.1 g) intravenously twice a day. The timeline of stroke aggravation and intervention is shown in Figure 4.

Fourteen days after antibiotic treatment, the clinical symptoms of the patient did not improve remarkably, with a serum RPR of 1:16. The patient was discharged and visited a venereal disease hospital for further treatment.

## Discussion

Syphilis, caused by *T. pallidum*, is a sexually transmitted disease. Syphilis can invade many organs, including the central nervous system. Neurosyphilis, including meningovascular syphilis, parenchymatous syphilis, syphilitic meningomyelitis, tabes dorsalis, general paresis, and gummas, can occur during any disease stages (1).

The invasion of *T. pallidum* in the central nervous system may cause immune cell aggregation and subsequent immune responses. Following invasion by spirochetes, lymphocytes, plasma cells, and other immune cells are infiltrated into the meninges and meningeal vessels. Subsequently, the cerebral arteries and brain parenchyma can be affected, causing parenchymatous syphilis and meningovascular syphilis. Heubner arteritis, mainly affecting the medium or large arteries, is characterized by intimal fibroblastic proliferation, medial thinning, adventitial inflammation, and fibrosis (5). Nissl-Alzheimer arteritis mainly involves the small vessels and is characterized by adventitial and intimal thickening (5, 6). Arterial stenosis or occlusion caused by syphilitic arteritis may lead to ischemic stroke (7).

Accurate diagnosis of neurosyphilis is difficult due to the wide range of potential clinical symptoms. It has been reported that stroke, as the first symptom, is found in 14.09% of individuals with neurosyphilis, while meningovascular syphilis accounted for most neurosyphilis cases (8). It is also difficult to differentiate neurosyphilis from an ischemic stroke during the early disease period. In this case, the patient first presented with stroke and multiple stenoses in the cerebral arteries. The common risk factors for stroke were absent, except for impaired glucose tolerance. We believe that the elevated blood glucose levels alone were not sufficient to explain such severe arterial stenosis. HR-MRI was performed to determine other possible causes. On HR-MRI, arteritis normally presents with concentric enhancement, which is segmental, uniform, and circular, and encloses the border of the artery with homogeneous signal intensity. In contrast, atherosclerotic stenosis tends to present with eccentric enhancement with irregular and heterogeneous wall thickening. In contrast, reversible vasoconstriction syndrome presents as diffuse, uniform, continuous wall thickening and enhancement with less signal intensity (9, 10).

In our case, concentric vessel wall enhancement in the left MCA was observed. The entire M1 segment of the left MCA and the A1 segment of the left ACA were involved, suggesting a high possibility of arteritis. Previous studies have reported similar concentric enhancement in the BA due to syphilitic arteritis (3, 4). Concentric enhancement on HR-MRI may help identify syphilitic arteritis. Infarction of the left basal ganglia was observed in our case, which was nourished by the lenticulostriate arteries. The lenticulostriate arteries were perforating arteries originating from the MCA and ACA. We propose that the abnormality of the vessels caused by arteritis in the left MCA and ACA destroyed the orifice of the lenticulostriate arteries, leading to ischemic lesions in the left basal ganglia. Arteritis in the lenticulostriate arteries might have also existed in the present case, although it was difficult to observe on radiological images. Both large and small arteries can be affected. Heubner arteritis and Nissl-Alzheimer arteritis can also occur concomitantly. Pathological examination may provide valuable information regarding the affected arteries. Multiple stenoses, including the right ACA, MCA, and BA, were observed in this case, while the enhancement of the affected vessel wall was not obvious. Similar stenosis has also been reported in other studies, and the reasons may be the inactive phases of arteritis or concomitant atherosclerosis (11, 12). Considering that the infarct area of the left basal ganglion could be explained by the blockage of the left lenticulostriate arteries, whereas no severe infarct was identified in the right hemisphere, syphilitic arteritis-induced blood flow arrest may account for the necrosis of certain brain areas. The characteristics of syphilitic arteritis on HR-MRI are rarely reported in the literature. Therefore, our case provides valuable information regarding the radiological features of syphilitic arteritis.

No international diagnostic criteria for neurosyphilis have been proposed to date. A Chinese clinical guideline indicated that CSF protein level ≥0.5 g/L, leukocyte count >10 × 10^6^/L, and positive non-treponemal or treponemal may be indicative of a diagnosis of neurosyphilis (13). In the present case, CSF TPPA test results were positive, together with elevated CSF protein levels and leukocytes. However, the CSF RPR test results were negative, while both RPR and TPPA test results were positive in the serum. One potential explanation is that the non-treponemal test has a high specificity but low sensitivity. In contrast, the treponemal test has a high sensitivity but low specificity (2, 14). It is not reliable to use a single test to identify neurosyphilis. Both non-treponemal and treponemal tests of the serum and CSF should be performed.

The neurological symptoms of the patient deteriorated twice. In the local hospital, the syphilitic etiology was not identified, and only ordinary stroke therapy was administered. The second aggravation occurred during antisyphilis therapy. The patient was allergic to both penicillin and ceftriaxone; therefore, roxithromycin was administered instead. Erythromycin was orally administered. However, it was less effective and did not readily infuse the brain (1, 14). Diarrhea is a potential side effect of erythromycin use, which may cause dehydration and hypoperfusion. Roxithromycin was then replaced with doxycycline. Another possible reason for the second aggravation may be hemorrhagic transformation. Antiplatelet therapy was administered initially. Most cases of meningovascular syphilis present with stroke (8) and many specialists use antiplatelet regimens (3, 7). However, there are no recommendations (7, 14–17). Intracerebral hemorrhage in neurosyphilis is rarely reported (18, 19). Antiplatelet therapy and reperfusion may increase the risk of hemorrhagic transformation. Some previous studies have reported that meningovascular syphilis causes not only arterial stenosis but also aneurysmal dilation or dissection, which may rupture leading to hemorrhage (19). The administration of antiplatelet therapy in neurosyphilis should be judiciously considered.

This case has several implications for the future management of neurosyphilis presenting with stroke. (1) HR-MRI findings of neurosyphilis have rarely been reported. This case provides the enhancement patterns of neurosyphilis arteritis on HR-MRI. (2) Antiplatelet medication should be judiciously administered since there is a potential risk of hemorrhagic transformation. Our study had some limitations. (1) Pathological examination was not performed because the patient declined examination. (2) Follow-up HR-MRI is needed to better understand the dynamic changes in the enhancement patterns of neurosyphilis arteritis.

## Conclusion

This case report described a patient with neurosyphilis who initially presented with aggravated stroke. HR-MRI showed concentric enhancement in the internal cerebral artery, suggesting arteritis, which is helpful in differentiating neurosyphilis from other cause-induced strokes. Antiplatelet medication should be used judiciously for neurosyphilis-related stroke.

## Data Availability Statement

The original contributions presented in the study are included in the article/supplementary material, further inquiries can be directed to the corresponding author/s.

## Ethics Statement

The studies involving human participants were reviewed and approved by the Human and Research Ethics committees of the First Hospital of Jilin University. The patients/participants provided their written informed consent to participate in this study.

## Author Contributions

KZ: organization and drafting and review of the manuscript. FC: review and critique of the manuscript. CW: review of the manuscript and improvement of English expressions. MS and YY: conception, organization, execution of the manuscript, and review and critique of the manuscript. All authors contributed to the article and approved the submitted version.

## Conflict of Interest

The authors declare that the research was conducted in the absence of any commercial or financial relationships that could be construed as a potential conflict of interest.

## Publisher's Note

All claims expressed in this article are solely those of the authors and do not necessarily represent those of their affiliated organizations, or those of the publisher, the editors and the reviewers. Any product that may be evaluated in this article, or claim that may be made by its manufacturer, is not guaranteed or endorsed by the publisher.
